# Determination of Causative Human Papillomavirus Type in Tissue Specimens of Common Warts Based on Estimated Viral Loads

**DOI:** 10.3389/fcimb.2020.00004

**Published:** 2020-01-24

**Authors:** Vesna Breznik, Kristina Fujs Komloš, Lea Hošnjak, Boštjan Luzar, Rajko Kavalar, Jovan Miljković, Mario Poljak

**Affiliations:** ^1^Department of Dermatology and Venereal Diseases, University Medical Centre Maribor, Maribor, Slovenia; ^2^Institute of Microbiology and Immunology, Faculty of Medicine, University of Ljubljana, Ljubljana, Slovenia; ^3^Institute of Pathology, Faculty of Medicine, University of Ljubljana, Ljubljana, Slovenia; ^4^Department of Pathology, University Medical Centre Maribor, Maribor, Slovenia; ^5^Faculty of Medicine, University of Maribor, Maribor, Slovenia

**Keywords:** common warts, human papillomaviruses, prevalence, viral load, causality

## Abstract

**Background:** Assessment of human papillomavirus (HPV) type-specific viral load (VL) is a valid tool for determining the etiology of HPV-related skin tumors, especially when more than one HPV type is detected within one lesion.

**Methods:** The causative HPV type was determined in 185 fresh-frozen tissue specimens of histologically confirmed common warts (CWs) collected from 121 immunocompetent patients. All tissues were tested using the type-specific quantitative real-time polymerase chain reactions (PCR) for the most common wart-associated *Alpha*-PV (HPV2/27/57) and *Mu*-PV types (HPV1/63/204). The presence of 23 additional low-risk HPVs was evaluated using a conventional wide-spectrum PCR.

**Results:** HPV DNA was detected in 176/185 (95.1%) CWs and multiple HPV types in 71/185 (38.4%) lesions. Using the VL approach and a robust cutoff of one viral copy/cell established in this study, HPV2/27/57 were determined as causative agents in 41/53 (77.3%) and 53/71 (74.7%) CWs with single and multiple HPVs, respectively.

**Conclusions:** CWs are mostly etiologically associated with HPV2/27/57 and only rarely with HPV1. In the majority of CWs containing multiple HPVs, a single HPV type was present in high concentration, indicating etiological association. No significant differences in VLs of lesion-causing HPV types in CWs containing single or multiple HPVs were found.

## Introduction

Cutaneous warts are ubiquitous benign skin tumors (van Haalen et al., 2009; de Koning et al., 2015), clinically presented as single or multiple dome-shaped keratotic lesions with exophytic growth (common warts; CWs) or endophytic keratotic papules on pressure points of soles (plantar warts) or smooth-surfaced flat-topped papules on the face and dorsum of hands (plane warts) (Jablonska et al., 1985; Cardoso and Calonje, 2011; Hogendoorn et al., 2018). Because cutaneous warts can be clinically misdiagnosed as corn, callus, fibroma, seborrheic keratosis, lichen ruber planus, molluscum contagiosum, anogenital wart, or even squamous cell carcinoma and melanoma (De Giorgi and Massi, 2006; Bae et al., 2009; Sondermann et al., 2016), skin biopsy followed by histological assessment is considered the diagnostic gold standard (Aldabagh et al., 2013).

Based on large population studies, which were mostly performed on swabs of warts' surfaces, cutaneous warts have been associated with various papillomavirus (PV) types belonging to genera *Alpha-, Gamma-, Mu-*, and *Nupapillomavirus (Alpha-, Gamma-, Mu-*, and *Nu-*PVs) (Schmitt et al., 2011; Bruggink et al., 2012; Al Bdour et al., 2013; de Koning et al., 2015) with the type spectrum varying across different anatomical regions. Whereas, CWs have usually been linked with infection with human PV (HPV) types HPV1, 2, 4, 7, 27, and 57, plantar warts are usually associated with HPV1, 4, 57, 60, 63, 65, and 66, and plane warts with HPV3, 10, 26–29, and 41 (Cardoso and Calonje, 2011). However, de Koning et al. recently showed the presence of wart-associated HPVs in up to 80% of swab samples of clinically normal skin (de Koning et al., 2015), thus raising the question of whether HPV types detected on warts' surfaces represent colonization, latent or productive HPV infection. In contrast to skin swabs, tissue specimens of cutaneous warts can provide information about HPVs present throughout the entire thickness of the epidermis (Aldabagh et al., 2013).

To the best of our knowledge, only seven small-scale HPV prevalence studies performed on a total of 129 histologically confirmed CW tissue samples have been published in peer-reviewed literature to date (Supplementary Table 1) (Gross et al., 1982; Chen et al., 1993; Egawa, 1994; Lei et al., 2009; Sun et al., 2010; de Koning et al., 2011; Šterbenc et al., 2017), with HPV2/27/57 and HPV1 being the most prevalent HPVs detected, but with extremely variable detection rates obtained in different studies (0–83.5 and 0–46.7% prevalence, respectively). Similarly, detection rates of multiple HPVs (the presence of more than one HPV type within one lesion) were tremendously variable (0–88.9%), prompting the development of tools for assessment of the most probable lesion-causative HPV among those detected within a particular CW (Šterbenc et al., 2017). Reliable information on causative HPV types vs. bystander HPVs in CWs is of great importance for informed decisions concerning targeted HPV types in future vaccines against cutaneous HPVs (Senger et al., 2009, 2010; Vinzón et al., 2014; Gaiser et al., 2015), which would be most beneficial for immunocompromised patients, including organ/tissue transplant recipients, often suffering from numerous and treatment-resistant cutaneous warts (Harwood et al., 1999; Bouwes Bavinck et al., 2007).

Köhler et al. (2009) suggested HPV type-specific viral load (VL) estimation as a surrogate marker for determining the etiology of CWs. Their study included eight dermoscopically but not histologically confirmed CW tissue samples of immunocompetent patients, and the VLs of HPV3, 27, and 57 were estimated at 5.1 × 10^1^, 1.1 × 10^4^, and 4.4 × 10^4^ viral copies/cell, respectively, suggesting an etiological role of these HPVs in a subset of CWs.

The aim of this study was to accurately determine the prevalence of several HPV types in by far the largest collection of fresh-frozen tissue samples of histologically confirmed CWs and to reliably assess the causative HPV based on estimation of type-specific HPV VLs using the robust cutoff value established in this study, especially in the most challenging warts containing multiple HPV types within one lesion.

## Materials and Methods

### Characteristics of Patients and Wart Samples

The study included 185 histologically confirmed fresh-frozen CW tissue samples prospectively collected from 121 immunocompetent patients, older than 5 years, referred to a single dermatology outpatient clinic in Slovenia between February 2014 and April 2016.

The majority of patients (108/121; 89.3%) were older than 12 years, with a mean age of 29.3 years (standard deviation (SD) 16.4, median 25 years, range 5–78 years); 66/121 (54.5%) patients were males.

A single wart was collected from 76/121 (62.8%) patients and multiple warts from 45/121 (37.2%) patients: two, three, and five warts were collected from 28/121 (23.2%), 16/121 (13.2%), and 1/121 (0.8%) patients, respectively. Warts were collected from hands including wrists (*n* = 131), lower extremities including dorsal sides of feet (*n* = 26), upper extremities (*n* = 14), and head and neck region (*n* = 14). Mean duration of warts was 16 months (*SD* 21.1, median 12 months, range 1–180 months), with a mean wart diameter of 8.4 mm (*SD* 7.2, median 7.0 mm, range 2–60 mm).

After obtaining patients' written informed consent, their CWs were photographed and 3–4 mm punch biopsies were performed under local anesthesia. Wart samples were then bisected, with half of the sample immediately frozen and stored in liquid nitrogen at −197°C until HPV testing and the other half fixed in 4% neutral buffered formalin and further prepared for routine histological hematoxylin and eosin staining (Kocjan et al., 2015). In cases in which a single patient had multiple warts, only warts that were more than 1 cm apart were sampled in order to reduce potential cross-contamination (de Koning et al., 2011; Tom et al., 2016).

The study was conducted in compliance with the Declaration of Helsinki and approved by the Medical Ethics Committee of the Republic of Slovenia (consent number 63/10/13).

### Detection of HPV Infection and HPV Typing

Total DNA from fresh-frozen CW tissue samples was extracted using the QIAamp DNA Mini Kit (Qiagen, Hilden, Germany), following protocol for nucleic acid purification from mammalian tissues, and spectrophotometrically quantified using the NanoDrop 2000c spectrophotometer (Thermo Fisher Scientific, Wilmington, DE). The integrity of extracted DNA was verified by real-time polymerase chain reaction (RT-PCR) amplification of a 268-bp fragment of human beta-globin gene, as described previously (Jancar et al., 2009). In order to determine the presence of HPV, 100 ng of each DNA sample was first tested for the presence of HPV2, 27, and 57, using type-specific quantitative multiplex RT-PCR, allowing sensitive detection and differentiation of HPV2, 27, and 57 in a single PCR reaction (Hošnjak et al., 2016). HPV2/27/57 RT-PCR–negative samples were additionally tested using a consensus primer set (Odar et al., 2014) in combination with Sanger sequencing, preferentially allowing detection of 23 low-risk *Alpha*-PVs (LR-HPVs): HPV2, 3, 6, 7, 10, 11, 13, 27, 28, 29, 32, 40, 42, 43, 44, 55, 57, 74, 77, 91, 94, 117, and 125 etiologically associated with various mucosal and cutaneous warts. Furthermore, in all samples, the determination of potential *Mu*-PV (HPV1, 63, and 204) infection was performed using three type-specific quantitative RT-PCRs, as described previously (Šterbenc et al., 2017). Type-specific VLs, expressed as ratios between the number of viral copies and human diploid cells, were estimated based on concentrations obtained with type-specific quantitative RT-PCRs and the quantitative 150-bp beta-globin RT-PCR, respectively (Šterbenc et al., 2017).

### Statistical Analysis

Results were presented as frequencies and percentages of descriptive nominal variables. For numerical variables, the minimum, maximum, arithmetic mean, standard deviation, and median values were calculated.

## Results

The 268-bp fragment of human beta-globin gene was successfully amplified from all 185 CW tissue samples, indicating adequate sample quality for further HPV testing.

As shown in Table 1, HPV DNA was detected in 176/185 (95.1%) samples, with single and multiple HPV infections detected in 105/185 (56.8%) and 71/185 (38.4%) samples, respectively. Two HPV types within a single lesion (double HPV infection) were present in one-third of CWs (66/185; 35.7%), and 1 in 50 lesions contained three different HPVs (5/185; 2.7%; Table 1). Overall, 12 different HPVs were identified in CWs, with HPV57, 27, and 2 representing by far the most common individual HPVs detected. Moreover, in combination with HPV1, these three HPVs were also the most prevalent in CWs containing two HPV types (Table 1).

**Table 1:** Distribution of human papillomavirus (HPV) types in 185 common warts with single and multiple HPV infections.

**HPV types detected within** **particular lesion, *n* (%)**	**HPV type(s)**	**Samples (*n*)**
Single HPV type: 105 (56.8)	HPV1	8
	HPV2	11
	HPV3	1
	HPV7	3
	HPV10	2
	HPV27	28
	HPV57	48
	HPV63	3
	HPV117	1
Two HPV types: 66 (35.7)	HPV1 + HPV2	4
	HPV1 + HPV7	2
	HPV1 + HPV10	2
	HPV1 + HPV27	15
	HPV1 + HPV29	1
	HPV1 + HPV57	31
	HPV1 + HPV63	2
	HPV1 + HPV94	1
	HPV1 + HPV117	1
	HPV7 + HPV57	1
	HPV27 + HPV57	3
	HPV57 + HPV63	2
	HPV57 + HPV204	1
Three HPV types: 5 (2.7)	HPV1 + HPV2 + HPV27	1
	HPV1 + HPV10 + HPV63	1
	HPV1 + HPV27 + HPV57	1
	HPV1 + HPV27 + HPV63	1
	HPV1 + HPV57 + HPV204	1
Warts negative for all HPVs tested: 9 (4.8)		
Total warts: 185 (100.0)		

As shown in Table 2, overall, the most frequent HPVs in CWs were HPV57, 1, 27, and 2, which were present in 67.7/185 (36.6%), 39.2/185 (21.2%), 38.0/185 (20.5%), and 13.3/185 (7.2%) CWs, respectively. The overall detection rate of HPV2/27/57 was 64.3% (119/185). In CWs with multiple HPVs, based on proportional attribution estimate (Insinga et al., 2008) HPV1 was the most frequent HPV type, detected in 31.2/71 (43.9%) samples, followed by HPV57, 27, and 63, detected in 19.7/71 (27.7%), 10/71 (14.1%), and 2.7/71 (3.8%) samples tested, respectively.

**Table 2:** Prevalence of human papillomavirus (HPV) types in 185 common warts of immunocompetent patients.

	**HPV type**	**Cumulative** ***n* = 185 (100%)**	**Single HPVs** ***n* = 105 (100%)**	**^a^Multiple HPVs** ***n* = 71 (100%)**
*Alphapapillomavirus 2*	HPV3	1 (0.5)	1 (1.0)	0
	HPV10	3.3 (1.8)	2 (1.9)	1.3 (1.8)
	HPV29	0.5 (0.3)	0	0.5 (0.7)
	HPV94	0.5 (0.3)	0	0.5 (0.7)
	HPV117	1.5 (0.8)	1 (1.0)	0.5 (0.7)
*Alphapapillomavirus 4*	HPV2	13.3 (7.2)	11 (10.5)	2.3 (3.3)
	HPV27	38 (20.5)	28 (26.7)	10 (14.1)
	HPV57	67.7 (36.6)	48 (45.7)	19.7 (27.7)
*Alphapapillomavirus 8*	HPV7	4.5 (2.4)	3 (2.8)	1.5 (2.2)
*Mupapillomavirus 1*	HPV1	39.2 (21.2)	8 (7.6)	31.2 (43.9)
*Mupapillomavirus 2*	HPV63	5.7 (3.1)	3 (2.8)	2.7 (3.8)
*Mupapillomavirus 3*	HPV204	0.8 (0.4)	0	0.8 (1.1)

a *To estimate the HPV type-specific prevalence in 71 warts with multiple HPV infections, the proportional attribution estimate was used. It was calculated using the method of Insinga et al. (2008), in which fractional allocation for each distinct HPV type was made*.

In the first part of the type-specific HPV VL study, 53 CWs containing a single HPV type were randomly selected to establish a robust cutoff value for further study on more challenging lesions containing multiple HPVs. As shown in Table 3, in these 53 randomly selected CWs containing a single HPV, the median VLs of HPV2, 27, and 57 were estimated at 3.3 × 10^4^, 3.2 × 10^4^, and 1.2 × 10^4^ viral copies/cell, respectively, and were significantly higher compared to the estimated VLs of HPV1 (8.2 × 10^−3^ viral copies/cell) and HPV63 (1.5 × 10^−3^ viral copies/cell). Based on the clear bimodal distribution of estimated HPV VLs in 53 randomly selected CWs containing a single HPV, ranging from 5.0 × 10^−4^ to 7.1 × 10^6^ copies/cell (Supplementary Table 2), a cutoff value of one viral copy/cell was used to discriminate between causative and non-causative HPVs (HPV bystanders). Consequently, 10/53 (18.9%) CWs containing a single HPV type and VLs of <1 viral copy/cell were assigned as “warts without determined causative HPV” (Table 4). As shown in Table 4, the most frequent causative HPVs in CWs containing a single HPV type were HPV57, 27, and 2, with an overall prevalence rate of 77.3% (41/53), followed by HPV1 (2/53; 3.8%). HPV63 was not determined as a causative agent in any of the CWs tested containing single HPV infection.

**Table 3:** Estimated viral loads in 53 randomly selected warts with single human papillomavirus (HPV) infections.

	**No. of warts (*N =* 53)**	**Viral load (copies/cell)**				
		**Minimum**	**Maximum**	**Median**	**Mean**	***SD***
HPV2	8	2.5 × 10^−3^	1.4 × 10^5^	3.3 × 10^4^	4.7 × 10^4^	5.1 × 10^4^
HPV27	15	6.3 × 10^3^	2.5 × 10^5^	3.2 × 10^4^	6.6 × 10^4^	7.1 × 10^4^
HPV57	22	6.0 × 10^−4^	1.7 × 10^5^	1.2 × 10^4^	2.8 × 10^4^	3.9 × 10^4^
HPV1	5	5.0 × 10^−4^	7.1 × 10^6^	8.2 × 10^−3^	1.5 × 10^6^	3.2 × 10^6^
HPV63	3	1.1 × 10^−3^	1.8 × 10^−2^	1.5 × 10^−3^	6.9 × 10^−3^	9.6 × 10^−3^

*Because estimated viral loads were not normally distributed, the median is statistically more appropriate than the mean*.

**Table 4:** Determined causative HPV types in 53 randomly selected warts with single human papillomavirus (HPV) infections and 71 warts with multiple HPV infections based on estimated type-specific HPV viral loads and set cutoff value.

	**Single HPVs, *n* (%)** ***N* = 53 (100%)**	**Multiple HPVs, *n* (%)** ***N* = 71 (100%)**
HPV2	7 (13.2)	5 (7.1)
HPV27	15 (28.3)	13 (18.3)
HPV57	19 (35.8)	35 (49.3)
HPV1	2 (3.8)	3 (4.2)
Without determined causative HPV	10 (18.9)	15 (21.1)

As shown in Table 4, the most prevalent causative HPVs in CWs containing multiple HPV types were again HPV57, 27, and 2, with an overall prevalence rate of 53/71 (74.7%), followed by HPV1 (3/71; 4.2%). In addition, based on data from Supplementary Table 3, the median VLs of HPV2, 27, 57, 1, 63, and 204 in CWs containing multiple HPVs were estimated at 2.6 × 10^4^, 2.0 × 10^4^, 2.8 × 10^4^, 1.6 × 10^−2^, 7.2 × 10^−3^, and 5.7 × 10^−3^ viral copies/cell, respectively. The VLs of HPV2/27/57 ranged from 6.0 × 10^−4^ to 3.6 × 10^5^ viral copies/cell and in the great majority of CWs were higher than 100 viral copies/cell, whereas the VLs of HPV1/63/204 ranged from 7.0 × 10^−4^ to 1.6 × 10^2^ viral copies/cell and were generally lower than one copy/cell, except in five HPV1-positive warts. Consequently, as shown in Supplementary Table 3, a single HPV with 10^3^-10^8^-fold higher VLs in comparison to VLs of other HPVs detected within the same wart was present in 52/71 (73.2%) CWs, and such an HPV was securely assigned as the causative HPV. In 4/71 (5.6%) warts, two HPVs present in the same lesion had high VLs and were both co-assigned as potentially causative HPVs (HPV1+27 and HPV1+57 in two CWs each). The causative HPV could not be reliably determined in 15/71 (21.1%) CWs containing multiple HPVs due to the fact that: (i) the VLs of all HPVs detected within the lesion were far <1 viral copy/cell (six samples), or (ii) in addition to targeted HPVs with minute VLs, the CW also contained additional HPVs (HPV7, 10, 29, 94, or 117), for which we were unable to measure VLs due to the lack of appropriate methods (nine samples; Supplementary Table 3). Similar to 10 CWs containing single HPVs with minute VLs, these 15 CWs containing multiple HPVs were labeled as “warts without determined causative HPV.” Thus, even though HPV63 and HPV204 were indeed detected in 2.7/71 (3.8%) and 0.8/71 (1.1%) warts containing multiple HPVs, respectively, they were not assigned as causative HPVs in any of the CWs tested due to minute VLs.

The majority of CWs containing multiple HPVs were obtained from hands (49/71; 69.0%), and others were collected from legs (10/71; 14.1%), arms (8/71; 11.3%), and head (4/71; 5.6%). The most frequent causative HPV in all four anatomical regions was HPV57 and caused all four CWs collected from the head. Interestingly, all four CWs samples with co-assigned causative HPVs (HPV1+27 and HPV1+57 in two CWs each) were collected from hands.

Among 45 patients with multiple CWs sampled, complete concordance of HPVs detected was found in only 14 (31.1%). However, at least one common HPV was present across all warts obtained from the same individual in 40/45 (88.9%) patients. In addition, in all 21 patients in whom multiple warts were available and HPV VLs could be estimated in all CWs sampled, 100% agreement across assigned causative HPVs was found.

## Discussion

Although a broad range of HPV types and a relatively high frequency of multiple HPVs have previously been reported in CWs' tissue samples (Gross et al., 1982; Chen et al., 1993; Egawa, 1994; Lei et al., 2009; Sun et al., 2010; de Koning et al., 2011; Šterbenc et al., 2017), previous studies suffered from small size, use of suboptimal specimens (formalin-fixed paraffin-embedded tissues), and inability to reliably assign the causative HPV type, especially in CWs containing multiple HPVs.

In this study, performed on the largest sample collection of CWs unambiguously diagnosed by histopathological assessment, HPV DNA detection rate was 176/185 (95.1%), which is in line with similar studies, in which HPV positivity ranged from 64.3 to 100% (Gross et al., 1982; Chen et al., 1993; Egawa, 1994; Lei et al., 2009; Sun et al., 2010; de Koning et al., 2011; Šterbenc et al., 2017). High HPV DNA prevalence in this study was most probably due to the use of optimal quality samples (fresh-frozen tissue), highly sensitive HPV detection and genotyping methods (Hošnjak et al., 2016; Šterbenc et al., 2017), and a relatively wide spectrum of HPV types tested (*n* = 26). Multiple HPVs were detected in 71/185 (38.4%) CWs, which is substantially higher in comparison to the majority of similar studies (Gross et al., 1982; Chen et al., 1993; Egawa, 1994; Lei et al., 2009; Sun et al., 2010; de Koning et al., 2011). In agreement with other studies (Chen et al., 1993; Lei et al., 2009; Šterbenc et al., 2017), the majority of CWs with multiple HPVs contained two HPVs. LR-HPVs and *Mu*-PVs were concomitantly detected in the vast majority of CWs containing multiple HPVs (65/71; 91.5%), with the most frequent combinations being HPV1+27 and HPV1+57, in line with the findings of some previous studies (Lei et al., 2009; Šterbenc et al., 2017).

Although two previous studies showed that, in cutaneous warts containing multiple HPVs, HPV type-specific VL measurement could be useful for differentiating between productive HPV infection and clinically latent infection, a relatively small number of CWs have previously been evaluated using the VL approach (*n* = 8 and *n* = 9, respectively) (Köhler et al., 2009; Šterbenc et al., 2017). This study explored HPV type-specific VLs measured on the largest collection of CWs (*n* = 124), and significantly higher median VLs of HPV2/27/57 were observed in comparison to HPV1/63/204 (10^4^ copies/cell and 10^−3^ copies/cell, respectively), irrespective of single or multiple HPVs present in CWs. This phenomenon could be a consequence of different phylogenetic backgrounds of the species *Alphapapillomavirus 4* and the genus *Mu*-PV (Bernard et al., 2010). The estimated high median VLs of HPV27 and 57 in this study are in line with the results of Köhler et al. (2009) and might explain the high prevalence of HPV2/27/57 in CWs of this cohort (119/185; 64.3%) and in previous studies (Gross et al., 1982; Lei et al., 2009; Sun et al., 2010; de Koning et al., 2011) due to copious viral shedding in the environment with subsequent growth of new warts.

To the best of our knowledge, VL cutoff(s) that could be used to reliably differentiate between causative and bystander HPV types in CWs, especially in challenging cases such as CWs containing multiple HPVs, have not been established yet. Based on the VLs measured in 53 randomly selected CWs containing a single HPV type, and clear bimodal distribution of the VLs obtained, in this study the cutoff value for causal determination was set at one copy/cell. Using this approach, HPV57 has been identified as the most important CW-related HPV causing a significant proportion of CWs (35.8 and 50.7% CWs containing single and multiple HPVs, respectively). Three HPV types (HPV2/27/57) were determined to be causative HPVs in three-quarters of CWs irrespective of whether CWs contained single or multiple HPVs, whereas HPV1 was assigned as the causative HPV in ~4% of CWs, again irrespective of single/multiple HPV status. Similarly, a previous study performed on nine samples determined HPV2/27/57 and HPV1 to be causative HPVs in 6/9 (66.6%) and 1/9 (11.1%) CWs, respectively (Šterbenc et al., 2017). Based on our findings, HPV1—a classic skin HPV type—seems to be rarely causally involved in the development of CWs, in contrast to its prominent etiological role in plantar warts in children (Rübben et al., 1993; Bruggink et al., 2012; de Koning et al., 2015) and its frequent presence in clinically normal skin (de Koning et al., 2015). Similarly, HPV63 and 204 were present in only a small fraction of CWs, and were not assigned as causative HPVs in any of the CWs in this study due to minute VLs. Our results are consistent with previous studies, reporting frequent etiological association of HPV63 with plantar warts (Egawa et al., 1993a, 2015; Bruggink et al., 2013; de Koning et al., 2015) and its frequent presence in normal skin (de Koning et al., 2015), but not in swabs of CW surfaces (de Koning et al., 2015). Furthermore, HPV204, the last officially recognized *Mu*-PV (Kocjan et al., 2015), could be etiologically more important in skin/mucosal lesions other than CWs, preferably in the anogenital region (Šterbenc et al., 2017). In conclusion, the frequent presence of *Mu-*PVs in minute concentrations suggests that they mostly cause clinically latent infections and are present in tissue specimens of CWs as bystanders, not causative HPVs.

In 4/185 (2.2%) CWs, we assigned two co-causative HPVs. In these CWs, we most probably had colliding wart lesions, each associated with an individual causative HPV, as recently demonstrated in cervical intraepithelial neoplasia lesions using laser capture micro-dissection and HPV genotyping, under the “one virus, one lesion” concept (Quint et al., 2012).

In line with previously reported findings (de Koning et al., 2015), in this study a remarkable 100% agreement across all assigned causative HPVs in individuals was found in all 21 patients who had multiple warts and in whom HPV VLs could be determined in all CWs sampled, suggesting that a single HPV type might be implicated in the development of multiple CWs in an individual patient.

In this study, *Mu*-PVs and LR-HPVs were not detected in 9/185 (4.9%) CWs, and, in one-fifth of CWs containing either single or multiple HPVs, the causative HPV remained undetermined, suggesting that these CWs might be etiologically attributed to HPV types from other species such as *Alphapapillomavirus* 2 or 8*, Gamma*-PVs, *Nu*-PVs, or other novel, still-unidentified HPV types. Although the cumulative prevalence of HPVs from species *Alphapapillomavirus* 2 and 8 (HPV7, 10, 29, 94, and 117) was relatively low (11.6/185; 6.3%) and their VLs were not estimated in this study, these HPV types could potentially be considered as the causative HPV of warts in question because HPV1 and 63, co-detected in these CWs, were present in minute quantities. In previous studies performed on histologically confirmed CWs, the reported detection rate of HPV4/60/65 (genus *Gamma*-PV) and HPV41 (genus *Nu-*PV) varied between 0 and 100% (Gross et al., 1982; Chen et al., 1993; Egawa, 1994; de Koning et al., 2011; Šterbenc et al., 2017). However, the highest (100%) prevalence of *Gamma*-PVs in the study by Egawa (1994) could be attributed to selection bias due to histologically discerned type-specific cytopathogenic effects (CPEs), which have assisted in the assignment of possible causative HPV type(s) in cutaneous warts in the past (Jablonska et al., 1985; Egawa et al., 1993a,b; Egawa, 1994). Nowadays, histopathological assessment is not considered a reliable diagnostic method for differentiation between HPV types/genus; for this purpose, we need HPV genotyping methods (Kalantari et al., 2009).

Because the distribution of HPV types and VLs of wart-associated HPVs were found to be independent of immunosuppression (Rübben et al., 1993; Köhler et al., 2009), the findings of this study could also be of importance for immunocompromised patient populations, for whom potential future vaccine(s) against cutaneous HPVs would be most beneficial. In particular, our results reinforce the need for obligatory inclusion of virus-like particles (VLPs) against HPV2/27/57 in such vaccines, which are currently in the early phase of development (Senger et al., 2010; Gaiser et al., 2015; Huber et al., 2017).

The main limitation of this study is potential selection bias because our cohort might not be fully representative of the general population due to enrollment of patients referred to the tertiary level of dermatological care, possibly due to more treatment-resistant warts or clinically atypical lesions. In addition, considering the benign nature of CWs and potential adverse events of the biopsy, including pain, allergic reactions to local anesthetics, and scarring (Wahie and Lawrence, 2007), only patients who consented to skin biopsy were enrolled, which may have caused additional selection bias and, consequently, fewer warts from children and from facial location were obtained. Another important limitation of this study is that due to technical limitations we were able to reliably assign the causative HPV based on an estimation of type-specific HPV VLs only for the six of the most prevalent CW-associated HPVs: *Alpha*-PV types HPV2/27/57 and *Mu*-PV types HPV1/63/204. Although we identified another six *Alpha-*PV 2 and 8 types (HPV types 3, 7, 10, 29, 94, and 117) as potential causative HPVs of nine warts, due to the lack of appropriate methods we were unable to measure the VLs of these HPVs, and consequently these CWs remain without a reliably assigned causative HPV.

In conclusion, this study was performed on by far the largest collection of histologically confirmed fresh-frozen CWs and—based on assessment of the causative HPV using an estimation of type-specific HPV VLs and the robust cutoff value of one viral copy/cell established in this study—it reliably assigned the causative HPV type in almost 80% of the CWs tested, including challenging lesions containing multiple HPVs. HPV57 has been identified as the most important CW-related HPV, causing at least one-third of CWs and three HPV types (HPV2/27/57) as causative HPVs of three-quarters of CWs irrespective of whether CWs contained single or multiple HPVs. This study provides further evidence for informed decisions about selecting the most appropriate targets for future vaccine(s) against cutaneous HPV-related benign tumors.

## Data Availability Statement

All datasets generated for this study are included in the article/Supplementary Material.

## Ethics Statement

The studies involving human participants were reviewed and approved by Medical Ethics Committee of the Republic of Slovenia (consent number 63/10/13). Written informed consent to participate in this study was provided by the participants' legal guardian/next of kin.

## Author Contributions

MP, KF, JM, and VB designed the study. VB and KF organized the database and performed the statistical analysis. LH and KF performed laboratory testing. RK and BL performed histological assessment. VB drafted the first version of the manuscript. LH, KF, and MP drafted individual sections of the manuscript. MP produced the final version of the manuscript. All authors revised manuscript, read it, and approved the submitted version.

### Conflict of Interest

The authors declare that the research was conducted in the absence of any commercial or financial relationships that could be construed as a potential conflict of interest.

## Supplementary Material

Supplementary Table 1 Summary of previously published HPV prevalence studies performed on histologically confirmed tissue samples of common warts obtained from immunocompetent individuals.

Supplementary Table 2 Estimated viral loads (VLs) of human papillomavirus (HPV) types 1, 2, 27, 57, and 63 in 53 randomly selected common warts containing a single HPV type and assigned causative HPV type based on estimation of type-specific HPV VLs.

Supplementary Table 3 Estimated viral loads (VLs) of human papillomavirus (HPV) types 1, 2, 27, 57, 63, and 204 in 71 common warts containing multiple HPVs and assigned causative HPV type based on estimation of type-specific HPV VLs.

## References

[B1] Al Bdour S. Akkash L. Shehabi A. A. (2013). Detection and typing of common human papillomaviruses among Jordanian patients. J. Med. Virol. 85, 1058–1062. 10.1002/jmv.23519 23588732

[B2] Aldabagh B. Angeles J. G. Cardones A. R. Arron S. T. (2013). Cutaneous squamous cell carcinoma and human papillomavirus: is there an association? Dermatol. Surg. 39, 1–23. 10.1111/j.1524-4725.2012.02558.x 22928516 PMC3521067

[B3] Bae J. M. Kang H. Kim H. O. Park Y. M. (2009). Differential diagnosis of plantar wart from corn, callus and healed wart with the aid of dermoscopy. Br. J. Dermatol. 160, 220–222. 10.1111/j.1365-2133.2008.08937.x 19067694

[B4] Bernard H. U. Burk R. D. Chen Z. van Doorslaer K. zur Hausen H. de Villiers E. M. (2010). Classification of papillomaviruses (PVs) based on 189 PV types and proposal of taxonomic amendments. Virology 401, 70–79. 10.1016/j.virol.2010.02.002 20206957 PMC3400342

[B5] Bouwes Bavinck J. N. Euvrard S. Naldi L. Nindl I. Proby C. M. Neale R.. (2007). Keratotic skin lesions and other risk factors are associated with skin cancer in organ-transplant recipients: a case-control study in The Netherlands, United Kingdom, Germany, France, and Italy. J. Invest. Dermatol. 127, 1647–1656. 10.1038/sj.jid.5700776 17380113 PMC2478722

[B6] Bruggink S. C. de Koning M. N. Gussekloo J. Egberts P. F. Ter Schegget J. Feltkamp M. C.. (2012). Cutaneous wart-associated HPV types: prevalence and relation with patient characteristics. J. Clin. Virol. 55, 250–255. 10.1016/j.jcv.2012.07.014 22884670

[B7] Bruggink S. C. Gussekloo J. de Koning M. N. Feltkamp M. C. Bavinck J. N. Quint W. G.. (2013). HPV type in plantar warts influences natural course and treatment response: secondary analysis of a randomised controlled trial. J. Clin. Virol. 57, 227–232. 10.1016/j.jcv.2013.02.021 23518443

[B8] Cardoso J. C. Calonje E. (2011). Cutaneous manifestations of human papillomaviruses: a review. Acta. Dermatovenerol Alp Pannonica Adriat. 20, 145–154. 22131115

[B9] Chen S. L. Tsao Y. P. Lee J. W. Sheu W. C. Liu Y. T. (1993). Characterization and analysis of human papillomaviruses of skin warts. Arch. Dermatol. Res. 285, 460–465. 10.1007/BF00376818 8274034

[B10] De Giorgi V. Massi D. (2006). Images in clinical medicine. Plantar melanoma–a false vegetant wart. N. Engl. J. Med. 355:e13. 10.1056/NEJMicm055674 17005944

[B11] de Koning M. N. Khoe L. V. Eekhof J. A. Kamp M. Gussekloo J. Ter Schegget J.. (2011). Lesional HPV types of cutaneous warts can be reliably identified by surface swabs. J. Clin. Virol. 52, 84–87. 10.1016/j.jcv.2011.06.016 21798797

[B12] de Koning M. N. Quint K. D. Bruggink S. C. Gussekloo J. Bouwes Bavinck J. N. Feltkamp M. C.. (2015). High prevalence of cutaneous warts in elementary school children and the ubiquitous presence of wart-associated human papillomavirus on clinically normal skin. Br. J. Dermatol. 172, 196–201. 10.1111/bjd.13216 24976535

[B13] Egawa K. (1994). New types of human papillomaviruses and intracytoplasmic inclusion bodies: a classification of inclusion warts according to clinical features, histology and associated HPV types. Br. J. Dermatol. 130, 158–166. 10.1111/j.1365-2133.1994.tb02894.x 8123568

[B14] Egawa K. Delius H. Matsukura T. Kawashima M. de Villiers E. M. (1993a). Two novel types of human papillomavirus, HPV 63 and HPV 65: comparisons of their clinical and histological features and DNA sequences to other HPV types. Virology 194, 789–799. 10.1006/viro.1993.1320 8389082

[B15] Egawa K. Shibasaki Y. de Villiers E. M. (1993b). Double infection with human papillomavirus 1 and human papillomavirus 63 in single cells of a lesion displaying only an human papillomavirus 63-induced cytopathogenic effect. Lab Invest. 69, 583–588. 8246450

[B16] Egawa N. Egawa K. Griffin H. Doorbar J. (2015). Human papillomaviruses; epithelial tropisms, and the development of neoplasia. Viruses 7, 3863–3890. 10.3390/v7072802 26193301 PMC4517131

[B17] Gaiser M. R. Textor S. Senger T. Schädlich L. Waterboer T. Kaufmann A. M.. (2015). Evaluation of specific humoral and cellular immune responses against the major capsid L1 protein of cutaneous wart-associated alpha-Papillomaviruses in solid organ transplant recipients. J. Dermatol. Sci. 77, 37–45. 10.1016/j.jdermsci.2014.11.002 25439730

[B18] Gross G. Pfister H. Hagedorn M. Gissmann L. (1982). Correlation between human papillomavirus (HPV) type and histology of warts. J. Invest. Dermatol. 78, 160–164. 10.1111/1523-1747.ep12506324 6276473

[B19] Harwood C. A. Spink P. J. Surentheran T. Leigh I. M. de Villiers E. M. McGregor J. M.. (1999). Degenerate and nested PCR: a highly sensitive and specific method for detection of human papillomavirus infection in cutaneous warts. J. Clin. Microbiol. 37, 3545–3555. 10.1128/JCM.37.11.3545-3555.1999 10523550 PMC85688

[B20] Hogendoorn G. K. Bruggink S. C. de Koning M. N. C. Eekhof J. A. H. Hermans K. E. Rissmann R.. (2018). Morphological characteristics and human papillomavirus genotype predict the treatment response in cutaneous warts. Br. J. Dermatol. 178, 253–260. 10.1111/bjd.15758 28646591

[B21] Hošnjak L. Fujs Komloš K. Kocjan B. J. Seme K. Poljak M. (2016). Development of a novel multiplex type-specific quantitative real-time PCR for detection and differentiation of infections with human papillomavirus types HPV2, HPV27, and HPV57. Acta Dermatovenerol. Alp. Pannonica Adriat. 25, 65–71. 10.15570/actaapa.2016.20 28006878

[B22] Huber B. Schellenbacher C. Shafti-Keramat S. Jindra C. Christensen N. Kirnbauer R. (2017). Chimeric L2-based virus-like particle (VLP) vaccines targeting cutaneous human papillomaviruses (HPV). PLoS ONE 12:e0169533. 10.1371/journal.pone.0169533 28056100 PMC5215943

[B23] Insinga R. P. Liaw K. L. Johnson L. G. Madeleine M. M. (2008). A systematic review of the prevalence and attribution of human papillomavirus types among cervical, vaginal, and vulvar precancers and cancers in the United States. Cancer Epidemiol. Biomarkers Prev. 17, 1611–1622. 10.1158/1055-9965.EPI-07-2922 18628412 PMC2587113

[B24] Jablonska S. Orth G. Obalek S. Croissant O. (1985). Cutaneous warts. Clinical, histologic, and virologic correlations. Clin. Dermatol. 3, 71–82. 10.1016/0738-081X(85)90051-3 2850861

[B25] Jancar N. Kocjan B. J. Poljak M. Bokal E. V. (2009). Comparison of paired cervical scrape and tumor tissue samples for detection of human papillomaviruses in patients with cervical cancer. Eur. J. Gynaecol. Oncol. 30, 675–678. 20099503

[B26] Kalantari M. Garcia-Carranca A. Morales-Vazquez C. D. Zuna R. Montiel D. P. Calleja-Macias I. E.. (2009). Laser capture microdissection of cervical human papillomavirus infections: copy number of the virus in cancerous and normal tissue and heterogeneous DNA methylation. Virology 390, 261–267. 10.1016/j.virol.2009.05.006 19497607 PMC2753400

[B27] Kocjan B. J. Šterbenc A. Hošnjak L. Chouhy D. Bolatti E. Giri A. A.. (2015). Genome announcement: complete genome sequence of a novel Mupapillomavirus, HPV204. Acta Dermatovenerol Alp. Pannonica Adriat. 24, 21–23. 10.15570/actaapa.2015.7 26086163

[B28] Köhler A. Meyer T. Stockfleth E. Nindl I. (2009). High viral load of human wart-associated papillomaviruses (PV) but not beta-PV in cutaneous warts independent of immunosuppression. Br. J. Dermatol. 161, 528–535. 10.1111/j.1365-2133.2009.09297.x 19519829

[B29] Lei Y. J. Gao C. Wang C. Han J. Chen J. M. Xiang G. C.. (2009). Molecular epidemiological study on prevalence of human papillomaviruses in patients with common warts in Beijing area. Biomed. Environ. Sci. 22, 55–61. 10.1016/S0895-3988(09)60023-4 19462689

[B30] Odar K. Kocjan B. J. Hošnjak L. Gale N. Poljak M. Zidar N. (2014). Verrucous carcinoma of the head and neck - not a human papillomavirus-related tumour? J. Cell. Mol. Med. 18, 635–645. 10.1111/jcmm.12211 24350715 PMC4000115

[B31] Quint W. Jenkins D. Molijn A. Struijk L. van de Sandt M. Doorbar J.. (2012). One virus, one lesion–individual components of CIN lesions contain a specific HPV type. J Pathol. 227, 62–71. 10.1002/path.3970 22127961

[B32] Rübben A. Krones R. Schwetschenau B. Grussendorf-Conen E. I. (1993). Common warts from immunocompetent patients show the same distribution of human papillomavirus types as common warts from immunocompromised patients. Br. J. Dermatol. 128, 264–270. 10.1111/j.1365-2133.1993.tb00169.x 8385983

[B33] Schmitt M. de Koning M. N. Eekhof J. A. Quint W. G. Pawlita M. (2011). Evaluation of a novel multiplex human papillomavirus (HPV) genotyping assay for HPV types in skin warts. J. Clin. Microbiol. 49, 3262–3267. 10.1128/JCM.00634-11 21813725 PMC3165586

[B34] Senger T. Becker M. R. Schädlich L. Waterboer T. Gissmann L. (2009). Identification of B-cell epitopes on virus-like particles of cutaneous alpha-human papillomaviruses. J. Virol. 83, 12692–12701. 10.1128/JVI.01582-09 19793806 PMC2786864

[B35] Senger T. Schädlich L. Textor S. Klein C. Michael K. M. Buck C. B.. (2010). Virus-like particles and capsomeres are potent vaccines against cutaneous alpha HPVs. Vaccine 28, 1583–1593. 10.1016/j.vaccine.2009.11.048 20003923 PMC7416602

[B36] Sondermann W. Zimmer L. Schadendorf D. Roesch A. Klode J. Dissemond J. (2016). Initial misdiagnosis of melanoma located on the foot is associated with poorer prognosis. Medicine 95:e4332. 10.1097/MD.0000000000004332 27442685 PMC5265802

[B37] Šterbenc A. Hošnjak L. Chouhy D. Bolatti E. M. Oštrbenk A. Seme K.. (2017). Molecular characterization, tissue tropism, and genetic variability of the novel Mupapillomavirus type HPV204 and phylogenetically related types HPV1 and HPV63. PLoS ONE 12:e0175892. 10.1371/journal.pone.0175892 28426749 PMC5398564

[B38] Sun C. Chen K. Gu H. Chang B. Jiang M. (2010). Association of human papillomavirus 7 with warts in toe webs. Br. J. Dermatol. 162, 579–586. 10.1111/j.1365-2133.2009.09564.x 19863503

[B39] Tom L. N. Dix C. F. Hoang V. L. Lin L. L. Nufer K. L. Tomihara S.. (2016). Skin microbiopsy for HPV DNA detection in cutaneous warts. J. Eur. Acad. Dermatol. Venereol. 30, e216–e217. 10.1111/jdv.13548 26854066

[B40] van Haalen F. M. Bruggink S. C. Gussekloo J. Assendelft W. J. Eekhof J. A. (2009). Warts in primary schoolchildren: prevalence and relation with environmental factors. Br. J. Dermatol. 161, 148–152. 10.1111/j.1365-2133.2009.09160.x 19438464

[B41] Vinzón S. E. Braspenning-Wesch I. Müller M. Geissler E. K. Nindl I. Gröne H. J.. (2014). Protective vaccination against papillomavirus-induced skin tumors under immunocompetent and immunosuppressive conditions: a preclinical study using a natural outbred animal model. PLoS Pathog. 10:e1003924. 10.1371/journal.ppat.1003924 24586150 PMC3930562

[B42] Wahie S. Lawrence C. M. (2007). Wound complications following diagnostic skin biopsies in dermatology inpatients. Arch. Dermatol. 143, 1267–1271. 10.1001/archderm.143.10.1267 17938340

